# Monitoring of measurable residual disease by next‐generation sequencing in patients with acute myeloid leukaemia

**DOI:** 10.1111/bjh.70135

**Published:** 2025-09-03

**Authors:** Augustin Boudry, Laure Goursaud, Delphine Lebon, Florent Dumezy, Véronique Harrivel, Alexandre Boue‐Raguet, Romane Joudinaud, Alice Marceau‐Renaut, Sandrine Geffroy, Carine Hauspie, Jan Brijs, Laurène Fenwarth, Céline Berthon, Jean‐Pierre Marolleau, Martin Figeac, Claude Preudhomme, Nicolas Duployez

**Affiliations:** ^1^ Laboratory of Hematology, Biology and Pathology Center CHU Lille Lille France; ^2^ University of Lille, ULR 2694–Metrics Lille France; ^3^ Department of Hematology Claude Huriez Hospital, CHU Lille Lille France; ^4^ University of Lille, U1277‐Canther, INSERM Lille France; ^5^ Department of Hematology and Cell Therapy CHU Amiens‐Picardie Amiens France; ^6^ University of Picardie Jules Verne, EA4666–HEMATIM Amiens France; ^7^ Laboratory of Hematology CHU Amiens‐Picardie Amiens France; ^8^ University of Lille, US 41–UAR 2014–PLBS, CNRS, INSERM, CHU Lille, Institut Pasteur de Lille Lille France

**Keywords:** AML, MRD, NGS

## Abstract

Measurable residual disease (MRD) is a strong prognostic factor in acute myeloid leukaemia (AML). Next‐generation sequencing (NGS) offers promise but must distinguish true signal from background. We assessed MRD in 98 adult AML patients in first complete remission after intensive chemotherapy using a duplex unique molecular identifier (UMI)‐based NGS capture panel. Error reduction analysis showed up to a 20‐fold decrease in artefactual calls versus conventional sequencing. Linearity studies with serial dilutions confirmed accurate quantification down to 0.01% variant allele frequency. In this cohort, NGS‐MRD positivity did not significantly affect overall survival (OS) or relapse‐free survival (RFS) after one course of chemotherapy. However, NGS‐MRD positivity >0.1%, excluding *DNMT3A*, *TET2*, *ASXL1*, *IDH1* and *IDH2* mutations, was significantly associated with inferior outcomes after two courses (OS: hazard ratio [HR] = 3.04, *p* = 0.0173; RFS: HR = 2.83, *p* = 0.0097). Combining multiparameter flow cytometry (MFC‐MRD) with NGS‐MRD identified a double‐positive subgroup with particularly poor outcomes after the first course (OS: HR = 7.98, *p* < 0.001; RFS: HR = 7.87, *p* < 0.001). These findings underscore that duplex UMI‐based NGS is a sensitive, quantitative approach for MRD assessment in AML, offering prognostic information complementary to MFC‐MRD.

## INTRODUCTION

Acute myeloid leukaemia (AML) is a genetically heterogeneous haematological malignancy characterized by the sequential accumulation of genomic alterations, resulting in a complex and dynamic clonal architecture with multiple clones.^1^, ^2^ Despite advances in treatment, the survival rate of adults with AML remains poor, with only 24% of patients surviving beyond 5 years.^3^, ^4^ While approximately 70% of younger adult patients achieve complete remission (CR) following intensive chemotherapy, relapse is attributed to the persistence of leukaemic cells. Detection of these persistent cells at a level lower than morphology‐based assessments, reported as measurable residual disease (MRD), is a recognized critical prognostic factor in AML, enabling a better assessment of remission depth, improved risk stratification and earlier detection of relapse.^5^, ^6^, ^7^, ^8^, ^9^ Currently recommended approaches include multiparameter flow cytometry (MFC), which requires a high level of expertise,^10^, ^11^ and quantitative polymerase chain reaction (qPCR), which is limited to a small set of recurrent aberrations.^11^

Next‐generation sequencing (NGS) has revolutionized the detection of mutations and has become a standard in the diagnostic work‐up for AML patients. The intrinsic error rates of conventional NGS are estimated to be between 0.1% and 1%, which greatly limits its use as a tool for MRD assessment. Error correction strategies using unique molecular identifiers (UMIs) have emerged to address this issue. By attaching a unique barcode to each DNA fragment before PCR amplification, simplex UMIs allow for the identification and subsequent correction of PCR and sequencing errors, thereby reducing background noise and increasing the accuracy of variant calling.^12^ However, simplex UMIs fail to correct errors affecting only one of the two DNA strands composing a DNA fragment. Duplex UMI technology involves the independent tagging of both strands of each DNA fragment prior to amplification, enabling the formation of a duplex consensus sequence that corrects a substantial portion of the errors introduced during library preparation and sequencing.^13^, ^14^

Here, we assessed MRD using a duplex UMI‐based NGS capture panel (NGS‐MRD) and investigated its prognostic impact during treatment as a complement to MFC in a cohort of 98 adult AML patients in first CR treated with intensive chemotherapy.

## METHODS

### Patient characteristics and MRD sampling

Ninety‐eight patients diagnosed between 2019 and 2022 and registered in the HDF‐AML observatory (CNIL 2214454v0) were included in the present study. Somatic mutations at diagnosis were profiled with a custom capture panel (Twist Bioscience, NovaSeq 6000, Supporting Information: Methods). Patient inclusion criteria were as follows: (i) achievement of first CR after two cycles of intensive chemotherapy, (ii) availability of bone marrow (BM) samples after the first and/or second course and (iii) presence of at least one somatic molecular marker for MRD evaluation. Core‐binding factor AML and acute promyelocytic leukaemia were excluded. The study complied with the Declaration of Helsinki and was approved by the Lille ethics committee and tumour bank (certification NF 96900‐2014/65453‐1).

### Targeted NGS‐MRD with duplex UMI

**FIGURE 1:**
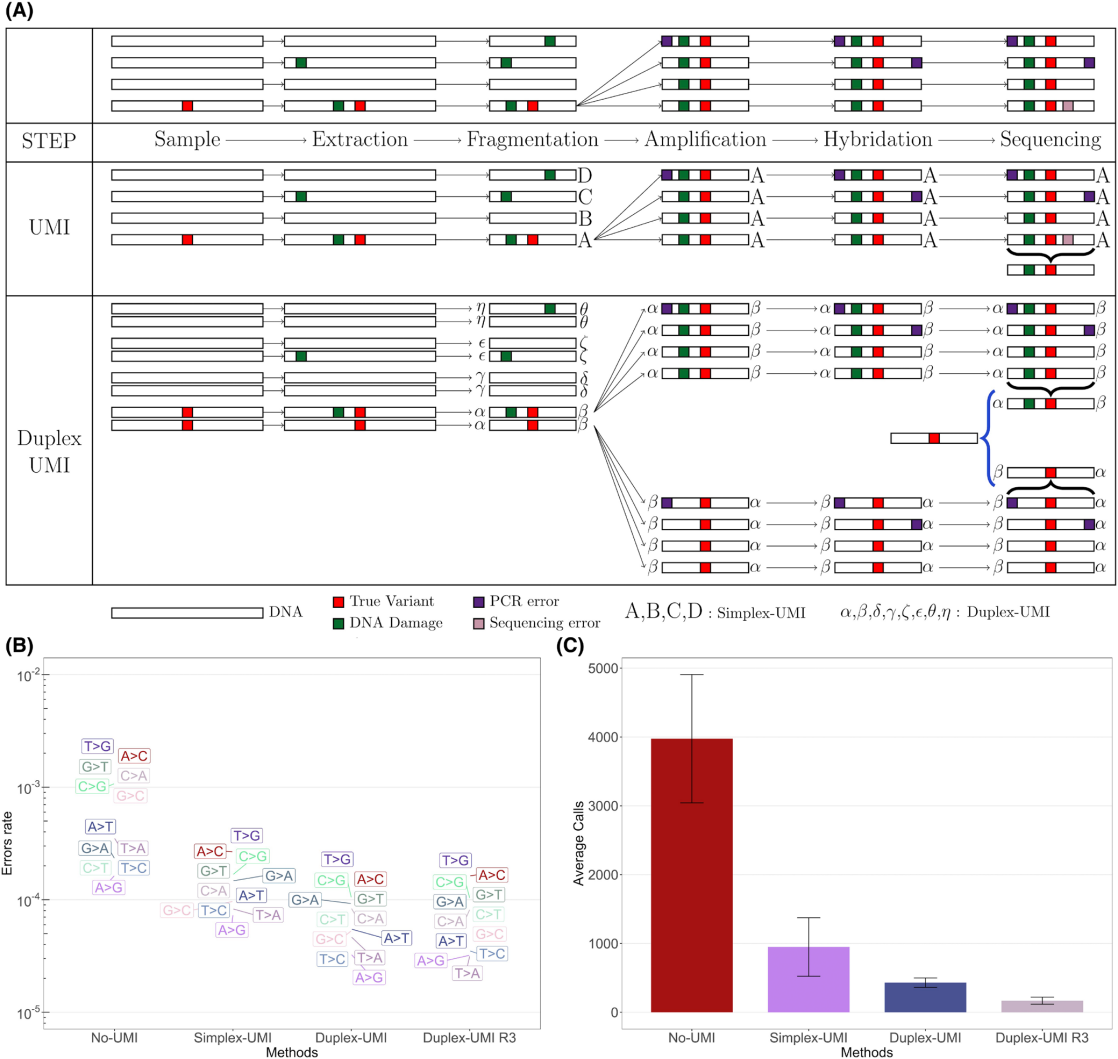
Principle and performance of next‐generation sequencing‐measurable residual disease (NGS‐MRD) with duplex unique molecular identifier (UMI). (A) Overview of UMI and duplex UMI usage (A, B, C, D refer to simplex UMIs; ⍺, β, δ, ɣ, ζ, ϵ, θ, η refer to duplex UMIs). (B) Comparison of error rates across methods. R3 refers to the call in three replicates (C) Evaluation of artefactual calls.

Genomic DNA was extracted by standard methods. For each sample, four DNA replicates of 200 ng each were sequenced using the following methodology. Libraries were prepared with a 34‐gene panel according to the Twist NGS target enrichment solution (Twist® V2), incorporating Duplex UMI (Figure 1A, Table S1). Libraries were sequenced on a NovaSeq 6000 platform (Illumina®), using paired‐end sequencing (2 × 151 base pairs). After adjusting the tiling probes in the capture pool to promote even coverage of GC‐rich genes, the panel included 556 probes spanning a total of 49 544 base pairs of genomic DNA. The bioinformatics pipeline is described in the supplemental appendix (Supporting Information: Methods, Figure S1).

### Positive and negative NGS controls

To evaluate panel performance, error reduction and linearity, the Tru‐Q 7 Horizon Discovery® Reference Standard was used as a positive control. This control contains 26 variants with a minimum variant allele frequency (VAF) of 1% (all assessed by droplet digital PCR [ddPCR]) representing the extensive mutational heterogeneity observed in AML and its various sequence contexts (Table S2). A negative control was also used (Reference Genomic DNA 103, ThermoFisher®).

### Statistical analysis

Continuous data were described using summary statistics, including the number of patients (*n*), mean, standard deviation, median and interquartile range. Categorical data were summarized using frequencies and percentages, with percentages calculated based on the number of patients without missing data. Group comparisons used Fisher's exact test for categorical data and *t*‐tests or Wilcoxon tests for continuous data.

Overall survival (OS) was calculated from diagnosis to death or last follow‐up; relapse‐free survival (RFS) from CR to relapse, death or last follow‐up. Survival curves were generated by Kaplan–Meier and compared with the log‐rank test. Multivariate analysis employed Cox regression, with proportional hazards assessed via Schoenfeld residuals, using separate models for post‐course 1 (PC1) and post‐course 2 (PC2) MRD. All tests were two‐tailed with *p* < 0.05, performed in R 4.3.2.

## RESULTS

### Patients' characteristics

A total of 98 patients were studied. Their characteristics are summarized in Table S3. All of them received intensive chemotherapy, of whom 70 (71%) underwent allogeneic stem cell transplantation (allo‐SCT). MFC‐MRD was available in 74 (PC1) and 55 (PC2). Samples were not available for other patients. NGS‐MRD was available in 86 (PC1) and 68 (PC2), totalling 154 samples. *NPM1* transcripts were assessed in 65 samples by qPCR or ddPCR.

### Performance of the NGS‐MRD with duplex UMI approach

Each sample yielded a mean of 2.33 × 10^8^ reads (SD: 2.31 × 10^7^), with an average of 66 498 unique molecules per position for each sample (SD: 10 827) and an average family size of 4. According to the Poisson distribution, the theoretical limit of detection was estimated at approximately 1 in 20 000 mutant alleles (Supporting Information: Methods).

To evaluate different approaches for error rate reduction, four methods were tested on positive and negative controls: standard deep sequencing (No‐UMI), simplex UMI, duplex UMI and duplex UMI with three replicates (duplex UMI R3). After masking sites corresponding to germline polymorphisms and variants present in controls, we quantified errors and the number of variant calls.

Using standard deep sequencing without UMI as the reference, we observed an error reduction of approximately sixfold with simplex UMI, 10‐fold with duplex UMI and 11‐fold with duplex UMI employing three replicates (Figure 1B, Table S4). Standard deep sequencing, configured to detect low‐prevalence variants, registered an average of 3975 calls (SD: 932). In contrast, the use of simplex UMI reduced the number of calls to an average of 949 (SD: 425). Duplex UMI further decreased the number of artefactual calls to an average of 430 (SD: 68). Finally, a duplex UMI strategy with three replicates was employed to suppress stochastic errors. Theoretically, with a per‐replicate error rate of 10^−3^, the probability of the same error occurring in all three replicates is 10^−9^. This approach markedly outperformed the other methods, with an average of only 168 calls (SD: 51) (Figure 1C). However, it should be noted that the benefit of replicate sequencing is most pronounced for low‐VAF variants, which are also inherently more susceptible to sampling bias.

To evaluate the capacity of the duplex UMI panel to detect known mutations with highly variable VAFs, we performed two 10‐fold serial dilutions by mixing positive DNA control with negative DNA control, resulting in VAFs of the mutations ranging from 25% to 0.01%. This approach successfully detected all 26 tested variants. Moreover, linearity and accurate quantification of VAFs were observed across all dilutions, with coefficients of variation ranging from 0.005 to 0.216 depending on the variant (Figure S2).

### Mutational landscape in first CR

**FIGURE 2:**
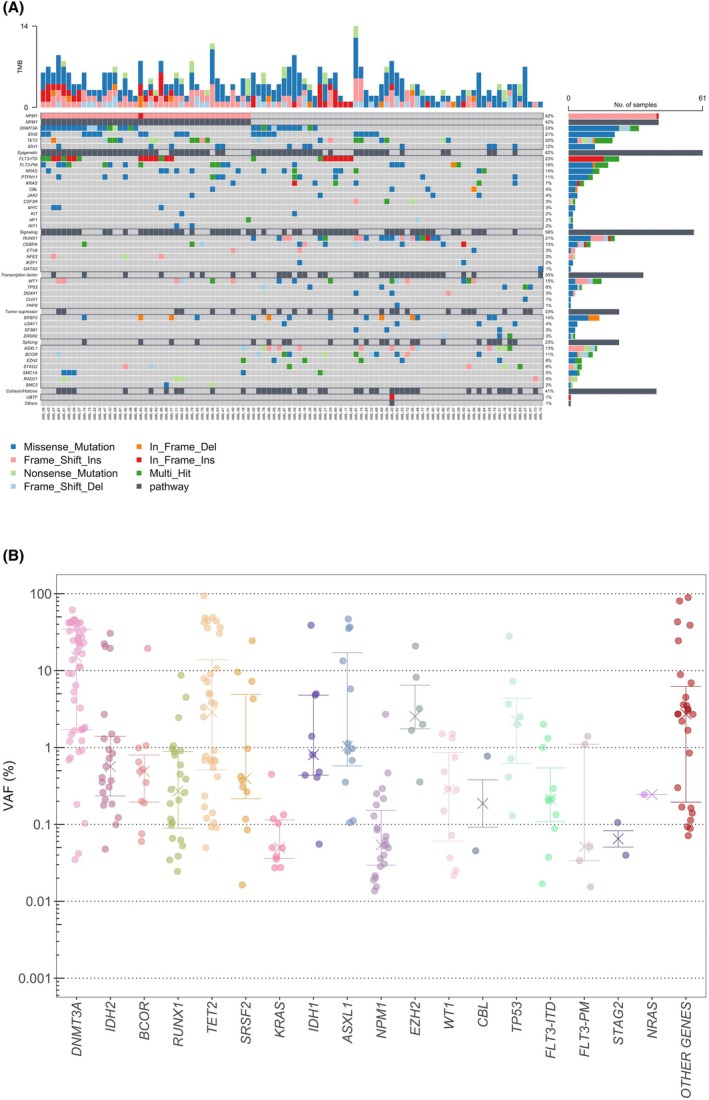
Mutational landscape and variant persistence in complete remission. (A) Oncoplot of baseline variants at AMLdiagnosis. (B) Variant allele frequencies plotted on a logarithmic scale for all detected mutations at MRD time points (post‐course 1 and post‐course 2) in AML patients in first CR. Each dot represents an individual mutation. Horizontal lines with crosshairs indicate the median VAF and interquartile ranges. AML, acute myeloid leukaemia; CR, complete remission; MRD, measurable residual disease; TMB, tumour mutational burden; VAF, variant allele frequency.

The mutational landscape of the studied cohort at diagnosis is shown in Figure 2A. In accordance with MRD definition, only baseline mutations already detected at diagnosis were tracked in remission samples (Supporting Information: Methods). In so doing, a total of 283 somatic mutations were detected by NGS‐MRD in 154 samples collected at PC1 and/or PC2. In PC1, 18 samples (21%) and, in PC2, 12 samples (18%) had no detectable mutation. When excluding DTA genes, these rates rose to 42 (49%) and 30 (44%) for PC1 and PC2 respectively. Among the positive samples (PC1 and PC2), the median number of persistent mutations per sample was 2 (range: 1–6). The median VAF was 0.77%, with significant variability across genes (Figure 2B). Overall, 11 genes (*ASXL1, BCOR, DNMT3A, EZH2, IDH1, IDH2, KRAS, RUNX1, SRSF2, TET2* and *TP53*) showed mutation persistence rates exceeding 50% in first CR. Higher VAFs (median >1%) were observed for *DNMT3A*, *TET2*, *TP53*, *EZH2* and *ASXL1*, consistent with their roles as early events and/or molecular predictors of poor molecular remission.

### Predictive impact of NGS‐MRD on outcome

**FIGURE 3:**
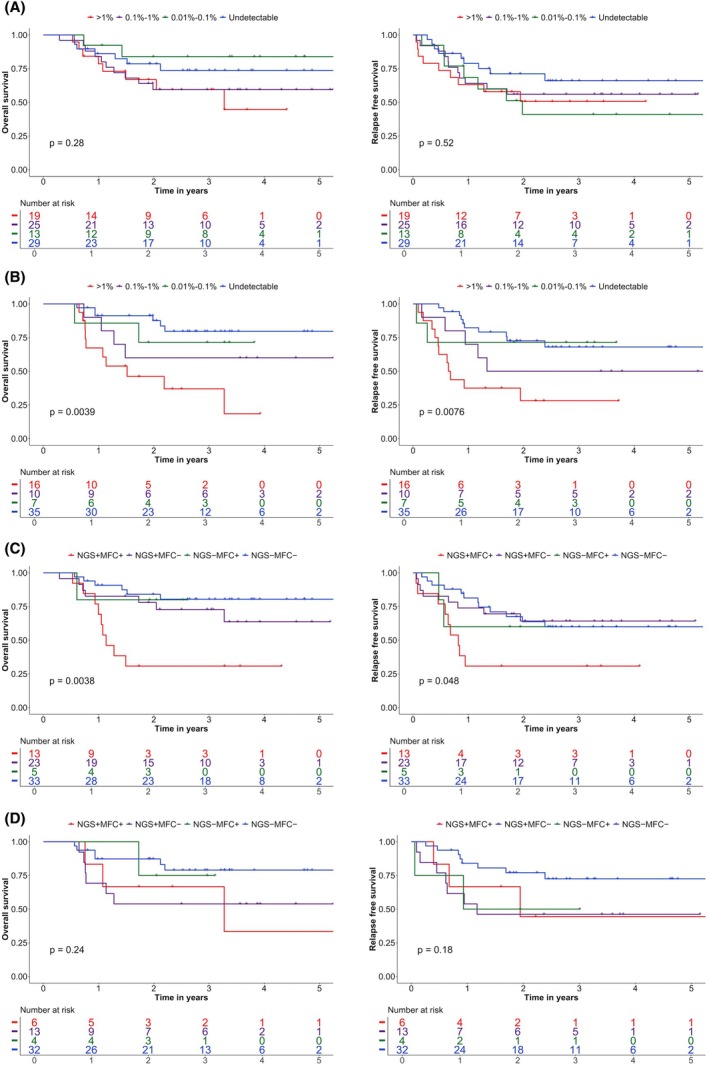
Outcomes of next‐generation sequencing‐measurable residual disease (NGS‐MRD) positivity after excluding *DNMT3A*, *TET2*, *ASXL1* and *IDH1/2* (DTAI) variants. (A) Overall survival (OS) and relapse‐free survival (RFS) at post‐course 1 (PC1). (B) OS and RFS at post‐course 2 (PC2). (C) Combined impact of NGS‐MRD and multiparameter flow cytometry (MFC‐MRD) on OS and RFS at PC1. (D) Combined impact of NGS‐MRD and MFC‐MRD on OS and RFS at PC2.

Different thresholds were tested for NGS‐MRD, including 1%, 0.1% and 0.01%, to assess their impact on patient outcomes. For samples harbouring multiple mutations, the highest VAF observed among their residual mutations was used as the representative VAF for MRD classification. NGS‐MRD positivity did not significantly influence OS or RFS at PC1, regardless of the threshold applied after exclusion of *DNMT3A*, *TET2*, *ASXL1*, *IDH1* and *IDH2* (DTAI) mutations (Figure 3A). In contrast, NGS‐MRD positivity was significantly associated with inferior OS and RFS at PC2, after exclusion of DTAI mutations (Figure 3B). The prognostic significance of a PC2‐positive NGS‐MRD emerged at the 0.1% threshold, with OS (*p* = 0.0024) and RFS (*p* = 0.0036) both adversely affected. Using a 1% cut‐off at PC2, NGS‐MRD positivity remained associated with poorer OS (*p* = 0.0005) and RFS (*p* = 0.00097). Outcomes of patients with NGS‐MRD levels between 0.01% and 0.1% were not statistically different from those who were MRD‐negative. Consequently, a threshold of 0.1% was adopted for subsequent analyses. NGS‐MRD positivity did not reach statistical significance either for PC1 or for PC2 when all mutations were included (Figure S3A,B) or when only DTA mutations were excluded (Figure S3C,D). Consequently, DTAI mutations were excluded from all subsequent analyses.

The majority of patients with positive NGS‐MRD at PC2 were classified within the European LeukemiaNet (ELN) adverse risk group (73%), while no significant differences were observed between NGS‐MRD positive and negative groups in terms of age at diagnosis, gender, white blood cell count, number of mutations at diagnosis, type of AML or allo‐SCT in first CR (Tables S5 and S6).

Multivariate analysis, adjusted for ELN 2022 risk classification, confirmed that NGS‐MRD positivity (>0.1%) at PC2 was an independent predictor of inferior outcome. The hazard ratio (HR) was 3.04 for OS (95 CI: 1.22–7.61; *p* = 0.0173) and 2.83 for RFS (95 CI: 1.29–6.24; *p* = 0.0097).

### Comparison between NGS‐MRD and standard MRD assays

*NPM1* mutant detection by NGS‐MRD (from genomic DNA) was compared to standard qPCR/ddPCR assays (from complementary DNA). NGS‐MRD positivity for *NPM1* mutant was defined as the detection of at least one consensus read in at least three replicates. Among the 65 samples studied by both methods, 47 (72%) were positive by NGS‐MRD and 45 (69%) were positive by qPCR/ddPCR. Concordant results were observed in 51 (78%) samples (39 and 12 samples were positive or negative by both methods respectively). In contrast, *NPM1* mutant MRD was detected only by NGS‐MRD in eight samples and only by qPCR/ddPCR in six samples (9.2%) (Figure S4A). The Spearman correlation coefficient between the two methods was 0.80.

MFC‐MRD and NGS‐MRD were compared using a threshold of 0.1% for NGS‐MRD. Among the 74 PC1 samples, 36 (48%) were positive by NGS‐MRD, compared with 18 (24%) positive cases by MFC‐MRD. Thirteen (17%) were positive by both techniques. Among the 55 PC2 samples, 19 samples (35%) were positive by NGS‐MRD, compared with 10 samples (18%) by MFC‐MRD. Six (11%) were positive by both techniques (Figure S4B).

### Combination of NGS‐MRD and MFC‐MRD to predict outcome

The combined impact of NGS‐MRD and MFC‐MRD status was evaluated at the 0.1% threshold, defining four groups: NGS+MFC+, NGS+MFC−, NGS−MFC+ and NGS−MFC−. At PC2, OS was 33%, 54%, 75% and 79% in these groups, respectively (*p* = 0.24), and RFS was 44%, 47%, 50% and 73% (*p* = 0.18), respectively, across 55 samples. In contrast, significant differences were observed at PC1: OS was 31%, 64%, 80% and 81% (*p* = 0.004), and RFS was 31%, 64%, 60% and 60% (*p* = 0.048) across 74 samples (Figure 3C,D). Patients in the NGS+MFC+ group at PC1 were predominantly classified in the adverse ELN risk category and more frequently underwent allo‐SCT in first CR. Other baseline characteristics, including age at diagnosis, gender, white blood cell count, number of diagnostic mutations and AML subtype, did not differ significantly between groups (Table S7). After adjustment for the ELN 2022 risk classification and allo‐SCT in first CR, NGS+MFC+ status at PC1 remained an independent predictor of reduced OS (HR = 7.98, *p* < 0.001) and RFS (HR = 7.87, *p* < 0.001; Table 1).

**TABLE 1:** Multivariate analysis of overall survival and relapse‐free survival based on combined NGS‐MRD and MFC‐MRD status at post‐course 1.

	*n*	OS			RFS		
		HR	95% CI	*p*‐value	HR	95% CI	*p*‐value
MRD group							
NGS−MFC−	33	—	—	—	—	—	—
NGS−MFC+	5	2.99	0.34, 26.7	0.3	3.72	0.75, 18.4	0.11
NGS+MFC−	23	3.57	1.08, 11.8	0.037	3.58	1.28, 9.98	0.015
NGS+MFC+	13	7.98	2.54, 25.1	<0.001	7.87	2.88, 21.5	<0.001
ELN2022 category							
Favourable	21	—	—	—	—	—	—
Adverse/Intermediate	53	6.61	1.41, 31.0	0.017	3.36	1.35, 8.33	0.009
Allo‐SCT first CR	41	0.10	0.04, 0.25	<0.001	0.05	0.02, 0.14	<0.001

Abbreviations: CR, complete remission; HR hazard ratio; MFC, multiparameter flow cytometry; MRD, measurable residual disease; NGS, next‐generation sequencing.

## DISCUSSION

In this study, we developed and evaluated an NGS capture panel incorporating duplex UMI technology for the sensitive detection of MRD in AML. First, our technique demonstrated excellent performance in terms of error reduction. By comparing standard deep sequencing, simplex UMI, duplex UMI and duplex UMI with three replicates, we showed that increasing the number of replicates and employing duplex correction substantially lowered artefactual variant calls. Our error suppression and artefact calling performance equals, and in some cases even exceeds reports using the single molecule molecular inversion probes (smMIP) method.^15^, ^16^ Moreover, our technique allows good linearity and overall limit of detection. These findings underscore the potential of this approach to detect rare persistent mutations with high quantitative precision.

Regarding *NPM1* mutations, we compared NGS‐MRD with RT‐PCR‐based MRD assays. While concordance was overall satisfactory, we observed some discordant cases. NGS‐MRD‐positive/RT‐PCR‐negative cases likely reflect differences in sample processing or RNA degradation, whereas RT‐PCR‐positive/NGS‐MRD‐negative MRD cases could be due to the different matrices used, given that *NPM1* is highly expressed at the RNA level. Importantly, all RT‐PCR‐positive/NGS‐MRD‐negative discordances occurred below the 2% decision threshold, as emphasized in the European LeukemiaNet consensus document on MRD in AML.^17^ Moreover, as noted by Vonk et al.,^18^ using an NGS‐based MRD assay targeting *NPM1* DNA offers the advantages of employing a stable input matrix, avoiding dependency on variable gene expression levels and detecting all *NPM1* mutation types rather than being limited to the most frequent *NPM1* variants.

Similarly, when comparing NGS‐MRD with MFC‐MRD, our data revealed some discrepancies, most frequently at PC1. The discordances between these methods likely reflect their inherent differences and are consistent with previous studies showing that molecular and MFC MRD assessments are complementary rather than interchangeable.^4^ Most *NPM1*‐mutated AML cases exhibited a CD34‐negative phenotype or monocytic differentiation features, which can be challenging to monitor by MFC‐MRD. Additionally, myelodysplasia‐related AML cases were frequently positive by NGS‐MRD but negative by MFC‐MRD.

Outcome analyses revealed that NGS‐MRD positivity at PC1 did not significantly affect OS or RFS, regardless of the threshold applied. However, at PC2, NGS‐MRD positivity was consistently associated with inferior OS and RFS across all thresholds tested. Notably, in our multivariate model adjusted for ELN 2022 risk classification, NGS‐MRD positivity at PC2 emerged as an independent predictor of adverse outcome. As suggested by CH. Tsai et al.,^19^ the PC2 time point may be more informative for risk stratification, potentially due to a more stable disease state and the cumulative impact of therapy.

Consistent with previous reports, our analysis revealed that mutations in DTA genes (*DNMT3A*, *TET2* and *ASXL1*) are frequently detected at high variant allele frequencies in MRD samples, suggesting that they likely originate from age‐related clonal haematopoiesis rather than representing true residual leukaemic burden.^16^, ^20^ Notably, *IDH1* and *IDH2* mutations have also been observed with similar characteristics, further complicating their utility as reliable MRD markers in CR.^21^, ^22^ In our cohort, NGS‐MRD positivity was significantly associated with inferior patient outcomes only when DTAI mutations were excluded. Thus, excluding DTAI mutations appears to improve the specificity of MRD assessment by better reflecting residual leukaemic disease rather than pre‐leukaemic clonal events. However, given our limited sample size, these results should be interpreted with caution, and further studies in larger cohorts are warranted before definitive conclusions can be drawn.

Furthermore, the combination of NGS‐MRD and MFC‐MRD analyses allowed us to identify a subset of patients with double‐positive (NGS+MFC+) results at PC1, who exhibited particularly poor outcomes in terms of OS and RFS even after adjustment for ELN risk. This joint application of molecular and flow MRD detection underscores the potential of a complementary approach to improve risk stratification in AML and early intervention, a strategy that warrants further investigation in larger cohorts. The lack of significant findings at PC2 may be partly attributed to the limited number of samples available for MFC analysis, highlighting the need for further studies with larger cohorts.

Despite these promising findings, our study has several limitations. The duplex UMI approach, while powerful, requires multiple replicates and entails longer library preparation times and higher costs compared with conventional assays.^16^, ^23^ Moreover, we did not compare our method with other approaches (e.g. smMIP, amplicon‐based methods with and without UMI) on the same samples, which would have allowed for a more balanced evaluation. For this study, we chose to adhere to a strict definition of MRD by monitoring only mutations that were already detected at diagnosis. Although technically feasible, the tracking of emerging mutations was not pursued due to the limited sample size. However, duplex UMI sequencing with three replicates reduces artefactual calls to levels comparable to standard diagnostic NGS without UMIs, indicating that de novo detection in MRD samples should be achievable using conventional filters. The small number of patients in our cohort may also limit the generalizability of our findings. Future studies involving larger and more diverse patient populations are necessary to validate our results, assess the impact of emerging mutations and refine the 0.1% threshold for MRD detection.

In conclusion, our study demonstrates that the use of a duplex UMI‐based NGS panel offers sensitive, quantitative and reproducible detection of MRD in AML. The integration of this approach with conventional cytometric methods provides complementary insights into disease dynamics and may ultimately guide more personalized treatment strategies. Further optimization and validation in larger cohorts will be essential to fully harness the clinical potential of this technology.

## AUTHOR CONTRIBUTIONS

C.P. and N.D. conceived and planned the experiments. A.B., F.D., V.H., R.J. and S.G. performed experiments. A.B., A.M.‐R., L.F., C.P. and N.D. interpreted the molecular data. A.B. and M.F. developed bioinformatics pipelines. L.G., D.L., A.B.‐R., J.B., C.B. and J.‐P.M. managed patients and provided clinical data. C.H. supervised the management of the database. A.B., M.F., C.P. and N.D. wrote the manuscript. All authors reviewed and approved the manuscript.

## FUNDING INFORMATION

L.F. is supported by the GIRCI/Cancéropole Nord‐Ouest (AAP‐AE‐2021; MARELAM project). A.B is supported by the Association Laurette Fugain (AO‐2024).

## CONFLICT OF INTEREST STATEMENT

The authors declare no potential conflicts of interest.

## Supporting information


Data S1.
