# Supplementary material for: Monitoring of measurable residual disease by next‐generation sequencing in patients with acute myeloid leukaemia
Source: Br J Haematol. 2025 Sep 3;207(5):1953–61. doi: 10.1111/bjh.70135 (PMC12624157; doi:10.1111/bjh.70135)
Supplement: Supplementary file 1 — Data S1. [file BJH-207-1953-s001.docx]

**Supplementary appendix:**

**Monitoring of measurable residual disease by next-generation sequencing in patients with acute myeloid leukaemia**

Augustin BOUDRY et al

# Supplementary methods

## Standard NGS at AML diagnosis

Genomic DNA was isolated using standard protocols. Libraries were prepared according to the Twist® NGS target enrichment solution (Twist BioScience®) following the manufacturer’s instructions and run on NovaSeq 6000® (Illumina®) with paired-end reads (2 x 151 bp) and a median read depth of 3,600X.

The bioinformatics pipeline was implemented as follows: BCL files produced by the sequencer were first converted to FASTQ format using bcl-convert (v4.2.4). The resulting FASTQ files were then quality-trimmed with fastp (v0.20.0). Next, the trimmed reads were aligned to the hg19 reference genome using bwa-mem (v0.7.17). Variant calling was performed with Mutect2 (v4.5.0.0) and VarDict. Variant allele frequencies (VAFs) were measured as the number of sequence reads matching a specific DNA variant divided by the overall coverage at that locus. Variants were named according to the Human Genome Variation Society nomenclature (GRCh37/hg19 build). Variant interpretation was performed considering minor allele frequencies (MAF) in the public gnomAD database of polymorphisms (variants with MAF > 0.02 in overall population/global ancestry or subcontinental ancestry were excluded) and VAFs. In addition, the FiLT3r algorithm (commit 4b64c9bb) was used for detection and precise quantification of *FLT3*-ITDs.

## MRD assessment by MFC (MRD-MFC)

MRD-MFC was prospectively performed on fresh bone marrow samples with Leukaemia-associated immunophenotype (LAIP) and different-from-normal (DfN) approaches. Briefly, the analysis was conducted using a two-tube panel, with each tube incorporating at least eight fluorescent markers. Both tubes used a backbone of CD34, CD38, CD45, and CD117 to identify haematopoietic progenitors. The first tube was further supplemented with antibodies against CD7, CD56, CD13, CD33, HLA-DR, and CD19. The second tube included additional markers—CD90 (Thy-1), a cocktail of CD97, CLL1, and TIM3, CD45RA, and CD123. For each tube, a minimum of 500,000 to 1,000,000 events were acquired.

## PCR-based MRD detection for NPM1 mutant transcript

Common *NPM1* mutant transcript levels (types A, B, and D) were prospectively quantified using a mutation-specific reverse transcriptase (RT)-qPCR assay as previously described (1). Rare *NPM1* mutant transcript levels were quantified by digital droplet PCR (ddPCR) using mutant-specific probes, as previously described (2). Transcript levels were normalized to *ABL1* copy number and expressed as a percentage (*NPM1*/*ABL1*).

## NGS-MRD: Bioinformatics analysis

The data generated were processed as follows. In summary, demultiplexing with UMI extraction was performed using bcl-convert (v4.2.4). FASTQ files were trimmed with fastp (v0.20.0). The trimmed FASTQ files were then aligned to the reference genome GRCh37 and deduplicated using the DRAGEN tool suite (v4.2). Singleton reads (originating from a single UMI) were discarded. The resulting deduplicated BAM files were analysed using Mutect2 (v4.5.0.0) and DRAGEN (v4.2) variant callers. In addition, FASTQ files were analysed using the FiLT3r algorithm (commit hash: 4b64c9bb) for *FLT3*-internal tandem duplications and *NPM1* indels (**Supplementary Figure 1**). A variant was considered positive if detected in at least three replicates. Only mutations already present at diagnosis were considered for NGS-MRD evaluation.

Detection of de novo variants in follow-up samples was outside the scope of this analysis; however, an analytical workflow is provided below:

- Germline exclusion: Discard variants with VAF ≈ 50% or 100%.
- Region and frequency filtering: Retain only exonic or splice-site variants with population frequency < 1% (gnomAD).
- Position-specific error threshold: Estimate background noise per site from a normal panel; accept only VAFs exceeding this noise.
- Read-support criteria: Require ≥ 10 alternate reads for SNVs and ≥ 3 for indels.
- Caller intersection: Report variants called by both Mutect2 and DRAGEN.
- Quality filters: Apply strand-bias filters, TLOD, and somatic quality score thresholds.
- COSMIC/diagnostic overlap: Prioritize variants present in COSMIC.
- Clonal coherence: Ensure variant VAFs and co-occurrence are consistent with the patient’s known clonal architecture.

## NGS-MRD: Theoretical limit of detection

To achieve a 95% probability of detection according to the Poisson distribution, the mean number of mutant copies ($\mu$) must be equal to 3 (i.e., $\mu= n \times p$). In our study, the average number of unique molecules obtained per panel is $n = 66,498$. To meet the condition $\mu= 3$, the probability p of detecting a mutation in a single unique molecule must be:

$$p=\frac{3}{66498}=\frac{1}{22166}\approx\frac{1}{20000}$$

Thus, the probability of detecting at least one mutant copy is given by:

$$P\left( X\geq1 \right)=1-e^{-\mu}=1-e^{-3}\approx0.95$$

This calculation demonstrates that, under our conditions, the theoretical limit of detection corresponds to approximately 1 mutant allele in 20,000.

## NGS-MRD: position-specific threshold

For all mutations identified at diagnosis across the patient cohort, forced variant calling was performed at MRD on all samples to capture even low-level variants. Outliers were excluded, and the noise level was quantified as the mean allele frequency plus three standard deviations for each possible variant. This combination of one-bp context and position-specific threshold ensures robust discrimination between true low-frequency variants and background noise.

# Supplementary figures

**Supplementary Figure 1:** Overview of the bioinformatic pipeline.

**Supplementary Figure 2:** Serial dilutions of positive controls with negative controls.


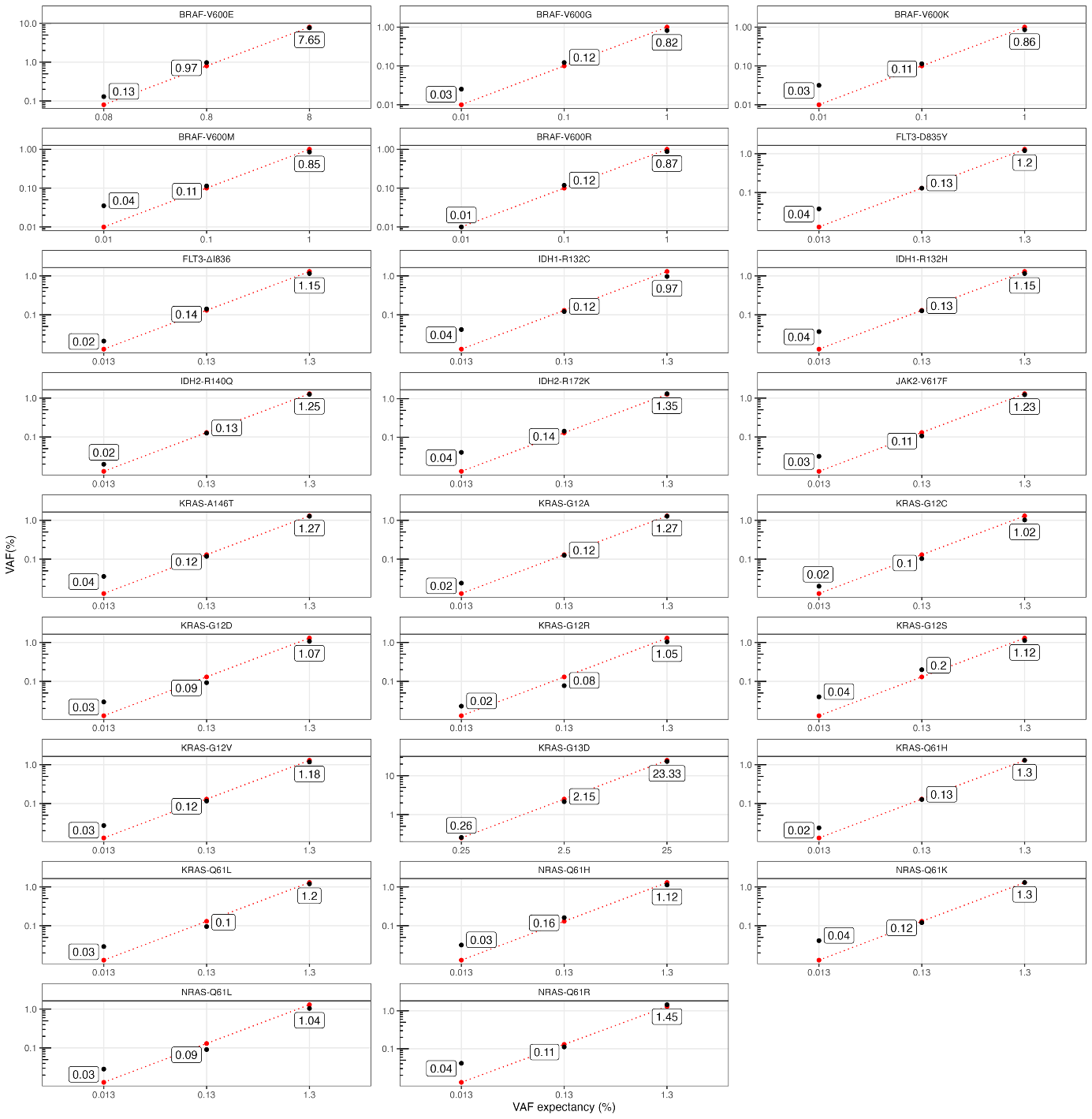


**Supplementary Figure 3:** Impact of NGS-MRD at different thresholds on OS and RFS. (A) all mutations included at post-course 1 (PC1). (B) all mutations included at post-course 2 (PC2). (C) *DNMT3A*, *TET2* and *ASXL1* (DTA) excluded at PC1. (D) DTA excluded at PC2.

**Supplementary Figure 4:** Comparison between NGS-MRD and standard MRD assays. (A) Detection of NPM1 MRD by NGS (from genomic DNA, y-axis) and PCR (from complementary DNA, x-axis). (B) Contingency table comparing NGS-MRD and MFC-MRD.

# Supplementary tables

**Supplementary Table 1:** Duplex-UMI-based NGS capture panel.

| Gene | Transcript | Target | Length (bp) | Number of probes |
| --- | --- | --- | --- | --- |
| ASXL1 | NM_015338 | E11-E12 | 3628 | 31 |
| BCOR | NM_001123385 | E02:E15 | 5548 | 52 |
| BRAF | NM_004333 | E11-E15 | 291 | 4 |
| CALR | NM_004343 | E09 | 226 | 2 |
| CBL | NM_005188 | E08-E09 | 517 | 6 |
| CEBPA | NM_004364 | E01 | 1136 | 29 |
| DDX41 | NM_016222 | E01:E17 | 2209 | 27 |
| DNMT3A | NM_022552 | E02:E23 | 3179 | 37 |
| EZH2 | NM_004456 | E02:E20 | 2636 | 31 |
| FLT3 | NM_004119 | E01:E24 | 3539 | 45 |
| HRAS | NM_005343 | E02:E04 | 578 | 6 |
| IDH1 | NM_005896 | E04-E07 | 484 | 5 |
| IDH2 | NM_002168 | E04-E07 | 353 | 4 |
| JAK2 | NM_004972 | E12-E14 | 266 | 3 |
| KRAS | NM_033360 | E02:E04 | 537 | 6 |
| MPL | NM_005373 | E10 | 117 | 1 |
| NF1 | NM_001042492 | E18 | 270 | 3 |
| NPM1 | NM_002520 | E11 | 88 | 4 |
| NRAS | NM_002524 | E02:E04 | 575 | 6 |
| PPM1D | NM_003620 | E06 | 578 | 5 |
| PTPN11 | NM_002834 | E03-E13 | 387 | 4 |
| RIT1 | NM_006912 | E05 | 215 | 2 |
| RUNX1 | NM_001754 | E02:E09 | 1603 | 18 |
| SF3B1 | NM_012433 | E13:E16 | 773 | 8 |
| SRSF2 | NM_003016 | E01 | 396 | 4 |
| SRY | NM_003140 | E01 | 203 | 2 |
| STAG2 | NM_001042749 | E03:E35 | 4467 | 61 |
| TET2 | NM_001127208 | E03:E11 | 6189 | 55 |
| TP53 | NM_001126112 | E02:E11 | 1443 | 18 |
| U2AF1 | NM_006758 | E02-E06 | 270 | 3 |
| UBA1 | NM_003334 | E03 | 101 | 1 |
| UBTF | NM_014233 | E10:E21 | 1890 | 21 |
| WT1 | NM_024426 | E01:E10 | 1769 | 19 |
| ZRSR2 | NM_005089 | E01:E11 | 1669 | 19 |
| Identity informative SNP | NA | NA | 1414 | 14 |
| Total |  |  | 49,544 | 556 |

SNP, single nucleotide polymorphism

**Supplementary Table 2:** List of variants represented in the positive control.

| Gene | Transcript | Variant | VAF (%) |
| --- | --- | --- | --- |
| BRAF | NM_004333 | V600E | 8 |
| BRAF | NM_004333 | V600G | 1 |
| BRAF | NM_004333 | V600K | 1 |
| BRAF | NM_004333 | V600M | 1 |
| BRAF | NM_004333 | V600R | 1 |
| FLT3 | NM_004119 | Δ836 | 1.3 |
| FLT3 | NM_004119 | D835Y | 1.3 |
| IDH1 | NM_005896 | R132C | 1.3 |
| IDH1 | NM_005896 | R132H | 1.3 |
| IDH2 | NM_002168 | R140Q | 1.3 |
| IDH2 | NM_002168 | R172K | 1.3 |
| JAK2 | NM_004972 | V617F | 1.3 |
| KRAS | NM_033360 | A146T | 1.3 |
| KRAS | NM_033360 | Q61H | 1.3 |
| KRAS | NM_033360 | Q61L | 1.3 |
| KRAS | NM_033360 | G12A | 1.3 |
| KRAS | NM_033360 | G12C | 1.3 |
| KRAS | NM_033360 | G12D | 1.3 |
| KRAS | NM_033360 | G12R | 1.3 |
| KRAS | NM_033360 | G12S | 1.3 |
| KRAS | NM_033360 | G12V | 1.3 |
| KRAS | NM_033360 | G13D | 25 |
| NRAS | NM_002524 | Q61H | 1.3 |
| NRAS | NM_002524 | Q61K | 1.3 |
| NRAS | NM_002524 | Q61L | 1.3 |
| NRAS | NM_002524 | Q61R | 1.3 |

VAF, variant allele frequency

**Supplementary Table 3:** Patients’ characteristics at AML diagnosis.

| Characteristic | Value |
| --- | --- |
| Total Patients | 98 |
| Age (y), median (Q1-Q3) | 58 (48-64) |
| Sex ratio (M:F) | 53:45 |
| WBC count |  |
| > 50 G/L | 20 (20%) |
| < 50 G/L | 78 (80%) |
| ELN 2022 risk classification |  |
| - Favourable | 26 (27%) |
| - Intermediate | 20 (20%) |
| - Adverse | 52 (53%) |
| Type of AML |  |
| - De novo | 86 (88%) |
| - Secondary | 12 (12%) |
| HSCT in first CR | 58 (59%) |

**Supplementary Table 4:** Details of error rates across methods

| Error Type | No-UMI | | Simplex-UMI | | Duplex-UMI | | Duplex-UMI R3 | |
| --- | --- | --- | --- | --- | --- | --- | --- | --- |
|  | **Mean** | **SD** | **Mean** | **SD** | **Mean** | **SD** | **Mean** | **SD** |
| A>C | 1.81E-03 | 1.54E-04 | 2.66E-04 | 1.35E-05 | 1.67E-04 | 1.22E-05 | 1.63E-04 | 1.19E-05 |
| A>G | 1.64E-04 | 3.70E-06 | 7.33E-05 | 2.56E-06 | 3.27E-05 | 4.41E-06 | 3.21E-05 | 3.39E-06 |
| A>T | 3.60E-04 | 5.13E-05 | 9.32E-05 | 4.28E-05 | 5.48E-05 | 3.81E-05 | 3.87E-05 | 3.02E-06 |
| C>A | 1.48E-03 | 5.82E-05 | 1.33E-04 | 4.59E-06 | 8.41E-05 | 4.47E-06 | 8.19E-05 | 3.80E-06 |
| C>G | 1.07E-03 | 6.31E-05 | 1.67E-04 | 4.81E-06 | 1.04E-04 | 5.81E-06 | 1.03E-04 | 6.35E-06 |
| C>T | 2.19E-04 | 6.13E-06 | 1.35E-04 | 7.28E-06 | 7.95E-05 | 1.04E-05 | 7.86E-05 | 8.98E-06 |
| G>A | 2.32E-04 | 2.05E-05 | 1.47E-04 | 2.17E-05 | 9.24E-05 | 2.51E-05 | 9.05E-05 | 2.24E-05 |
| G>C | 1.04E-03 | 6.63E-05 | 9.66E-05 | 2.35E-06 | 5.29E-05 | 3.53E-06 | 5.20E-05 | 2.83E-06 |
| G>T | 1.49E-03 | 5.46E-05 | 1.55E-04 | 5.50E-06 | 9.76E-05 | 5.58E-06 | 9.50E-05 | 4.43E-06 |
| T>A | 3.52E-04 | 5.41E-05 | 8.29E-05 | 4.22E-05 | 4.67E-05 | 3.68E-05 | 3.17E-05 | 3.30E-06 |
| T>C | 1.63E-04 | 7.03E-06 | 7.77E-05 | 6.82E-06 | 3.57E-05 | 8.55E-06 | 3.52E-05 | 7.99E-06 |
| T>G | 1.86E-03 | 1.57E-04 | 3.00E-04 | 1.80E-05 | 1.85E-04 | 1.45E-05 | 1.80E-04 | 1.21E-05 |
| Average | 5.80E-04 | 3.46E-05 | 1.30E-04 | 9.15E-06 | 7.48E-05 | 1.02E-05 | 6.92E-05 | 6.04E-06 |
|  |  |  |  |  |  |  |  |  |

**Supplementary Table 5:** Impact of NGS-MRD at 0.1% on OS and RFS at PC2

|  | Negative (n=42) | Positive (n=26) | p-value |
| --- | --- | --- | --- |
| OS | 78.16% | 41.33% | 0.0024 |
| RFS | 68.94% | 37.3% | 0.0036 |

**Supplementary Table 6:** Comparison of patients’ characteristics according to NGS-MRD status at post-course 2, with 0.1% threshold.

| Characteristic | Negative (n=42) *^1^* | Positive (n=26) *^1^* | p-value |
| --- | --- | --- | --- |
| Age | 55 (48,62) | 56 (42, 64) | 0.9 |
| Sex |  |  | 0.081 |
| - Female | 22 (52%) | 8 (31%) |  |
| - Male | 20 (48%) | 18 (69%) |  |
| ELN Risk Category |  |  | 0.03 |
| - Favourable | 16 (38%) | 2 (7.7%) | – |
| - Intermediate | 12 (29%) | 5 (19%) | – |
| - Adverse | 14 (33%) | 19 (73%) | – |
| WBC | 11 (3, 44) | 18 (5, 40) | 0.6 |
| Number of Mutations | 4 (3, 6) | 5 (3, 6) | 0.2 |
| Type of AML |  |  | 0.2 |
| - De novo | 40 (95%) | 22 (85%) |  |
| - Secondary | 2 (4.8%) | 4 (15%) |  |
| HSCT in first CR | 22 (52%) | 17 (65%) | 0.3 |

*^1^* Median (Q1, Q3); n (%)

**Supplementary Table 7:** Comparison of patients’ characteristics according to NGS-MRD and MFC-MRD status at post-course 1

| Characteristic | NGS-MFC-*^1^*  *(n=33)* | NGS-MFC+*^1^*  *(n=5)* | NGS+MFC-*^1^*  *(n=23)* | NGS+MFC+*^1^*  *(n=13)* | p-value |
| --- | --- | --- | --- | --- | --- |
| Age | 56 (47, 62) | 65 (59, 67) | 60 (54, 64) | 54 (39, 60) | 0.2 |
| Sex |  |  |  |  | 0.2 |
| - Female | 21 (64%) | 1 (20%) | 10 (43%) | 5 (38%) |  |
| - Male | 12 (36%) | 4 (80%) | 13 (57%) | 8 (62%) |  |
| ELN Risk Category |  |  |  |  | 0.007 |
| - Favourable | 16 (48%) | 1 (20%) | 3 (13%) | 1 (7.7%) |  |
| - Adverse/Intermediate | 17 (52%) | 4 (80%) | 20 (87%) | 12 (92%) |  |
| WBC | 7 (2, 44) | 21 (1, 62) | 8 (2,39) | 10 (4, 23) | >0.9 |
| Number of Mutations | 3 (3, 6) | 5 (2, 6) | 4 (3, 6) | 4 (2,5) | 0.8 |
| Type of AML |  |  |  |  | 0.5 |
| - De novo | 31 (94%) | 5 (100%) | 19 (83%) | 11 (85%) |  |
| - Secondary | 2 (6.1%) | 0 (0%) | 4 (17%) | 2 (15%) |  |
| HSCT in first CR | 12 (36%) | 3 (60%) | 17 (74%) | 9 (69%) | 0.023 |

*^1^* Median (Q1, Q3); n (%)

# References

1. Balsat M, Renneville A, Thomas X, de Botton S, Caillot D, Marceau A, et al. Postinduction Minimal Residual Disease Predicts Outcome and Benefit From Allogeneic Stem Cell Transplantation in Acute Myeloid Leukemia With NPM1 Mutation: A Study by the Acute Leukemia French Association Group. J Clin Oncol. 10 janv 2017;35(2):185‑93.

2. Lesieur A, Thomas X, Nibourel O, Boissel N, Fenwarth L, De Botton S, et al. Minimal residual disease monitoring in acute myeloid leukemia with non-A/B/D-NPM1 mutations by digital polymerase chain reaction: feasibility and clinical use. Haematologica. 1 juin 2021;106(6):1767‑9.
